# Supplementary figures and images for: Transcriptome sequencing and comparative analysis of adult ovary and testis identify potential gonadal maintenance-related genes in Mauremys reevesii with temperature-dependent sex determination
Source: PeerJ. 2019 Mar 8;7:e6557. doi: 10.7717/peerj.6557 (PMC6410691; doi:10.7717/peerj.6557)

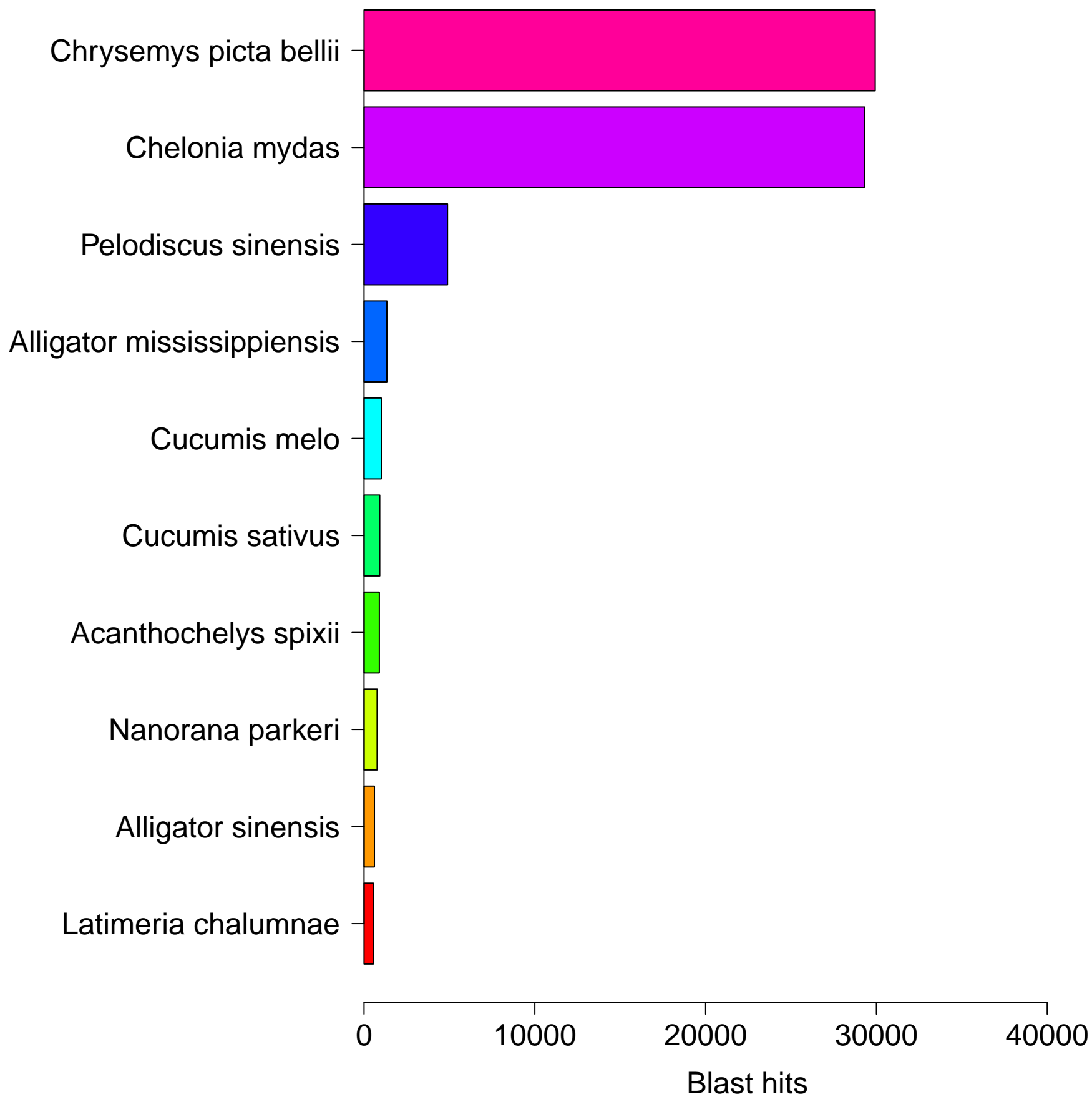

Supplement: Supplemental Information 7 [file peerj-07-6557-s007.pdf]

Gene Function Classification(GO)

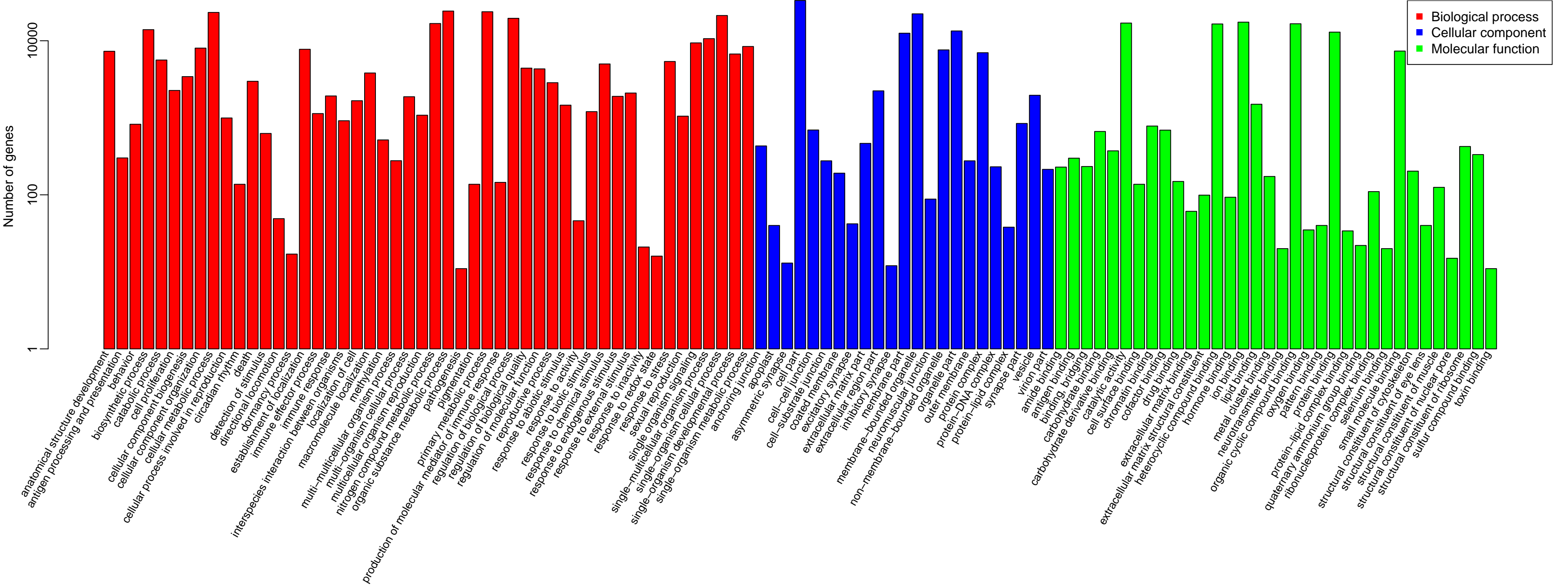

Supplement: Supplemental Information 8 [file peerj-07-6557-s008.pdf]
